# Conjoint propagation and differentiation of human embryonic stem cells to cardiomyocytes in a defined microcarrier spinner culture

**DOI:** 10.1186/scrt498

**Published:** 2014-09-15

**Authors:** Alan Tin-Lun Lam, Allen Kuan-Liang Chen, Jian Li, William R Birch, Shaul Reuveny, Steve Kah-Weng Oh

**Affiliations:** Stem Cell Group, Bioprocessing Technology Institute, Agency for Science, Technology and Research (A*STAR), Singapore, 138668 Singapore; Institute of Materials Research and Engineering, Agency for Science, Technology and Research (A*STAR), Singapore, 117602 Singapore

## Abstract

**Introduction:**

Myocardial infarction is accompanied by a significant loss of cardiomyocytes (CMs). Functional CMs, differentiated from human embryonic stem cells (hESCs), offer a potentially unlimited cell source for cardiac disease therapies and regenerative cardiovascular medicine. However, conventional production methods on monolayer culture surfaces cannot adequately supply the large numbers of cells required for such treatments. To this end, an integrated microcarrier (MC) bioprocessing system for hESC propagation and subsequent CM differentiation was developed.

**Methods:**

Production of hESC-derived CMs was initially established in monolayer cultures. This control condition was compared against hESC expansion on laminin-coated MC with cationic surface charge, in a stirred serum-free defined culture. Following expansion, the hESC/MC aggregates were placed in a CM differentiation medium, using Wnt signalling modulators in four different culture conditions. This process eliminated the need for manual colony cutting. The final optimized protocol was tested in stirred spinner flasks, combining expansion and differentiation on the same MC, with only media changes during the culture process.

**Results:**

In the propagation phase, a 15-fold expansion of viable pluripotent HES-3 was achieved, with homogeneous sized aggregates of 316 ± 11 μm. Of the four differentiation conditions, stirred spinner flask cultures (MC-Sp) provided the best controlled aggregate sizes and yielded 1.9 × 10^6^ CM/ml, as compared to 0.5 × 10^6^ CM/ml using the monolayer cultures method: a four-fold increase in CM/ml. Similar results (1.3 × 10^6^ CM/ml) were obtained with an alternative hESC H7 line. The hESC/MC-derived CM expressed cardiac-specific transcription factors, structural, ion channel genes, and exhibited cross-striations of sarcomeric proteins, thus confirming their cardiac ontogeny. Moreover, E-4031 (0.3 μM) prolonged the QT-interval duration by 40% and verapamil (3 μM) reduced it by 45%, illustrating the suitability of these CM for pharmacological assays.

**Conclusions:**

We have demonstrated a robust and scalable microcarrier system for generating hESC-derived CM. This platform is enabled by defined microcarrier matrices and it integrates cell propagation and differentiation within a continuous process, in serum-free culture media. It can generate significant numbers of CM, which are potentially suitable for future clinical therapies.

**Electronic supplementary material:**

The online version of this article (doi:10.1186/scrt498) contains supplementary material, which is available to authorized users.

## Introduction

Cardiovascular disease is a major cause of deaths worldwide [1]. Most of these diseases, such as ischemic heart disease and myocardial infarction, are associated with the permanent loss of heart muscle, in the form of functional cardiomyocytes (CMs) [2]. Given the limited intrinsic regenerative capacity of the mammalian heart, recent studies have focused on engineering the constituent cells for tissues that may potentially repair damaged cardiac muscle. Cells intended for clinical use need to be expanded easily in significant numbers and should differentiate into mature, fully functional CMs, capable of integrating to the damaged host tissue [3, 4]. Human pluripotent stem cells (hPSCs), such as human embryonic stem cells (hESCs) and human induced pluripotent stem cells, offer the opportunity of a promising therapeutic approach in which functional CMs generated *in vitro* can be transplanted into an injured heart and restore its function [4–6].

hPSCs have been differentiated with growth factor-based [7–10] or small molecule-based [11–15] differentiation protocols. Recently, a highly efficient CM differentiation protocol was reported by Lian and colleagues [12, 13]. The protocol uses two small molecules to modulate the Wnt signalling pathway, with early enhancement of differentiation at day 0 by 6-bromoindirubin-3′-oxime (BIO) or CHIR99021 and subsequent repression of the Wnt pathway, from day 3, by adding inhibitor of Wnt production IWP2 or IWP4 [12]. Up to 98% cardiac troponin T (cTnT)-positive functional human CMs was reported for monolayer cultures (MNL) [12].

Pluripotent hESCs have been generally differentiated in two different platforms either on tissue culture plates [16–20] or embryoid body (EB) cultures [21, 22]. The suspended EB cultures have the potential for volumetric scale-up [23, 24], which poses significant challenges in planar tissue culture plates [25]. However, the generation of EBs involves dissociating or cutting aggregate cultures and subsequent cell reaggregation [26]. These processes are labour intensive and can affect cell viability, making the process difficult to automate and scale up. Moreover, it is hard to control aggregate sizes and shapes, and such heterogeneity therefore affects differentiation reproducibility [27, 28]. Although EBs of controlled size can be formed by hanging drops [15] or forced aggregation methods [29, 30], they are limited to experiments on the scale of a research laboratory. In addition, high production costs, primarily generated by expensive growth factors [31], are not practical for large-scale manufacture. These hurdles must be overcome in developing an optimal method for production of large amounts of CMs as recently reviewed by our group [31].

Scalable methods for expansion of hPSCs on commercial microcarriers (MCs) coated with extracellular matrix proteins have been developed achieving high cell expansion (eightfold to 18-fold) in long-term, robust, suspended cultures [32–34]. hPSCs retain pluripotent markers, retain the ability to differentiate into the three primary germ layers, and exhibit normal diploid karyotypes. Recently, a defined polystyrene-based (≈100 μm diameter) MC coated with cationic poly-l-lysine (PLL) polyelectrolyte and laminin (LN) was developed by our group [35]. This defined matrix supports the expansion of HES3, H7, and IMR90 under continuous agitation from a single-cell seeding solution achieving high cell yields (14-fold, eightfold, and sevenfold cell expansion, respectively) with excellent viability (>90%). Importantly, uniform size (320 to 420 μm) hPSC/MC aggregates are generated during growth in agitated spinner flasks. These aggregates may be used as EBs for further differentiation into specific cell lineages, thus eliminating the need for cell dissociation, cutting, and reaggregation. Our group has similarly developed an integrated MC propagation and differentiation platform for neuroprogenitors, with yields 11.6-fold higher than those from EB culture [36].

**Figure 1:**
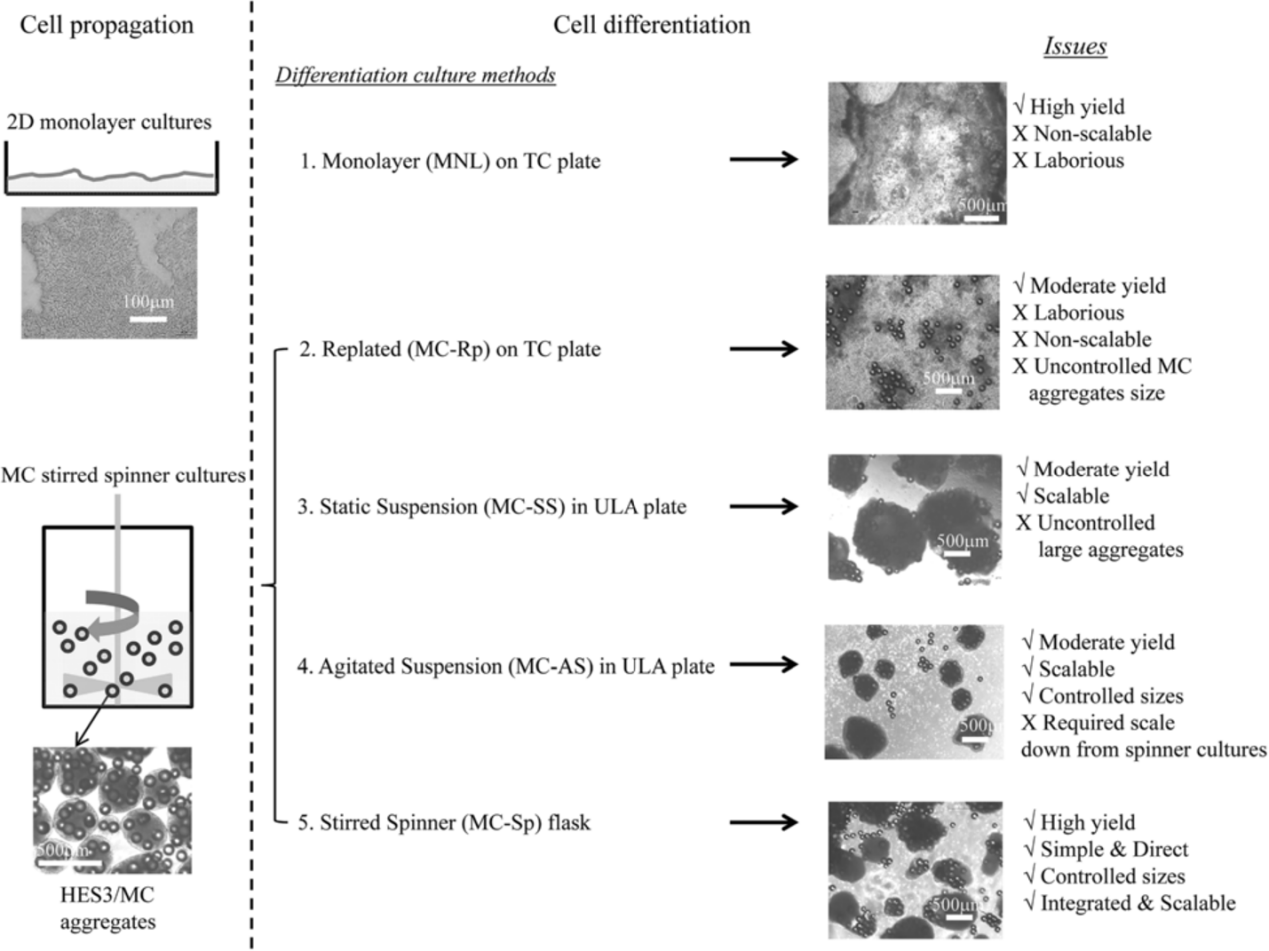
**Schematic presentation of the experimental plan to evaluate cardiomyocyte differentiation via monolayer and human embryonic stem cell/microcarrier aggregates cultures.** Morphological representation of the derived cardiomyocytes from the different culture regimes. Scale bars: 500 μm for human embryonic stem cell (hESC)/microcarrier (MC) aggregate cultures, and 100 μm for the two-dimensional (2D) monolayer hESC cultures. MC-AS, hESC/MC aggregates in agitated suspension cultures; MC-Rp, monolayer replated cultures; MC-Sp, hESC/MC aggregates in spinner cultures; MC-SS, hESC/MC aggregates in static suspension cultures; TC, tissue culture.

The present study implements uniform-sized aggregates, formed during stirred spinner culture using MCs coated with PLL + LN, to generate CMs within a continuous process, in the same culture vessel. The differentiation of these hESC/MC aggregates was directed to CMs using a differentiation protocol based on Wnt modulators [12, 13]. Five culture regimes were evaluated, ranging from benchmarking with MNL to MC-based cultures in static conditions and under agitation (Figure 1). The efficiency of cardiac differentiation was quantified and the molecular, structural, and functional properties of hESC/MC-derived CMs were also examined. This study provides a foundation for the scalable and robust production of hESC-derived CMs in large numbers, by means of a conjoint propagation and differentiation bioprocess in a defined environment, free of growth factors.

## Materials and methods

### Microcarriers and their coatings

Polystyrene MCs with an average diameter of 97 ± 10 μm were purchased from Thermo-Fisher Scientific (Waltham, MA, USA). These MC beads were sterilised by exposure to gamma irradiation (10 minutes, 10 kGray/hour) in a ^60^Co irradiator (Gammacell 200 Excel; Ottawa, ON, Canada) before being utilised for culture.

For coating with PLL (70 to 150 kDa; Sigma-Aldrich, St. Louis, MO, USA), 12 μl of 1 mg/ml PLL were added to 20 mg MC in 600 μl phosphate-buffered saline (PBS), to make a final PLL concentration of 20 μg/ml. This was incubated at 4°C overnight and then rinsed with PBS before further coating. Similarly, the coating of murine LN (Life Technologies, Carlsbad, CA, USA) was achieved by adding 20 μl of 1 mg/ml LN to 20 mg PLL-coated MC in 600 μl PBS, to make a final LN concentration of 33 μg/ml [35]. The MCs were similarly incubated at 4°C overnight, followed by a rinse with PBS before being used for cell culture.

### Culture of human embryonic stem cells

The HES3 ([46 X,X]; ES Cell International, Alameda, CA, USA) and H7 ([46 X,X]; WiCell Research Institute, Madison, WI, USA) cell lines were routinely maintained on Matrigel-coated plates using mTeSR™1 hESC medium (StemCell Technologies, Vancouver, BC, Canada) as described previously [35, 37]. Cultures were incubated at 37°C in a humidified atmosphere with 5% carbon dioxide. Unless otherwise stated, all culture media (for cell propagation and differentiation) and supplements were purchased from Life Technologies and all reagents and chemicals were purchased from Sigma-Aldrich.

### Human embryonic stem cell propagation in spinner cultures

hESC MC culture was run in a 50 ml spinner flask (BellCo, Vineland, NJ, USA), using a procedure similar to the one described previously [32, 35]. Briefly, hESC/MC aggregates from six-well ultra-low attachment plate (Costar, Tewksbury, MA, USA) cultures were mechanically dissociated into small cell clumps and seeded at 2 × 10^5^ cells/ml in the 50 ml spinner flask that contained 25 ml hESC medium and 200 mg PLL + LN-coated MC. The sample was incubated at 37°C/5% carbon dioxide for 24 hours in static conditions, after which another 25 ml hESC medium were added and the culture was then agitated at 30 rpm for 6 days. Eighty per cent of the spent medium was replaced daily with fresh hESC medium. The cell concentration and cell viability were determined daily using a Nucleocounter NC-3000 (Chemometec, Davis, CA, USA). Pluripotent markers were measured by flow cytometry on day 7. The size of the hESC/MC aggregates was measured from images taken using an Olympus IX70 microscope (Olympus, Shinjuku-ku, Tokoyo, Japan), with average dimensions determined using NIH image J software [35].

### Direct cardiomyocyte differentiation from propagated hESCs via temporal modulation of canonical Wnt signalling

To induce CM differentiation, the direct differentiation protocol from Lian and colleagues that uses small molecule, CHIR99021 (Selleck, Houston, TX, USA) and IWP2 (Stemgent, Cambridge, MA, USA), modulators of Wnt signalling was adopted [12, 13] and optimised (see Additional file 1). This was implemented in five culture regimes (Figure 1): MNL, replated hESC/MC aggregate cultures (MC-Rp), hESC/MC aggregates in static suspension cultures (MC-SS), hESC/MC aggregates in agitated suspension cultures (MC-AS), and hESC/MC aggregates in spinner cultures (MC-Sp). Contracting monolayers and beating aggregates were visualised daily under phase-contrast microscopy (Evos, AMG, Mill Creek, WA, USA). On day 20 of the differentiation protocol, cells from all cultures were harvested and analysed by fluorescence-activated cell sorting (FACS). The sizes of differentiated aggregates were measured using the Olympus IX70 microscope, and average sizes were determined using NIH image J software.

#### Culture regime 1: monolayer

A hESC single-cell suspension was obtained by dissociating confluent HES3 with TrypLE™ (Life Technologies). Viable cells (4 × 10^5^ cells/well) from the single cell suspension were seeded into a 12-well plate (Corning, Tewksbury, MA, USA) coated with 10 μg/ml LN. The cultures were maintained in mTeSR™1 hESC medium in a 37°C/5% carbon dioxide incubator for 3 to 4 days, until cells reached confluence. At this point, differentiation was started by removing spent hESC medium and adding RPMI/B27 medium without insulin (referred to as differentiation medium) and containing 12 μM CHIR99021 [12]. After 24 hours (that is, day 1 of differentiation), the medium was aspirated and replaced with fresh differentiation medium. On day 3 of differentiation, differentiation medium with 5 μM IWP2 was added. On day 5 of differentiation, the IWP2-containing differentiation medium was removed and the medium was subsequently refreshed every 2 days, until day 11. Cells were then maintained in differentiation medium with insulin until day 20.

#### Culture regime 2: replated hESC/MC aggregates

About 50 hESC/MC aggregates (equivalent to 4 × 10^5^ cells/well) from spinner flask cultures were inoculated onto a 10 μg/ml LN-coated 12-well plate. After incubation for 1 day to allow the aggregates to attach, the plate was washed with PBS to remove unattached aggregates. Direct differentiation was subsequently initiated by removing spent hESC medium and adding differentiation medium containing 15 μM CHIR99021 (day 0). After 24 hours the differentiation medium was replenished, and 10 μM IWP2 was added at day 3. This IWP2 was removed during the medium exchange on day 5. Cells were then maintained in differentiation medium with insulin from day 11 until day 20.

#### Culture regime 3: hESC/MC aggregates in static suspension

About 50 hESC/MC aggregates from spinner cultures were incubated in a 12-well ultra-low attachment plate (Nunc, Rochester, NY, USA) and directly subjected to cardiac differentiation simply by changing the hESC medium into CHIR99021-containing differentiation medium followed by IWP2 treatment, as described for MC-Rp above.

#### Culture regime 4: hESC/MC aggregates in agitated suspension

**Figure 2:**
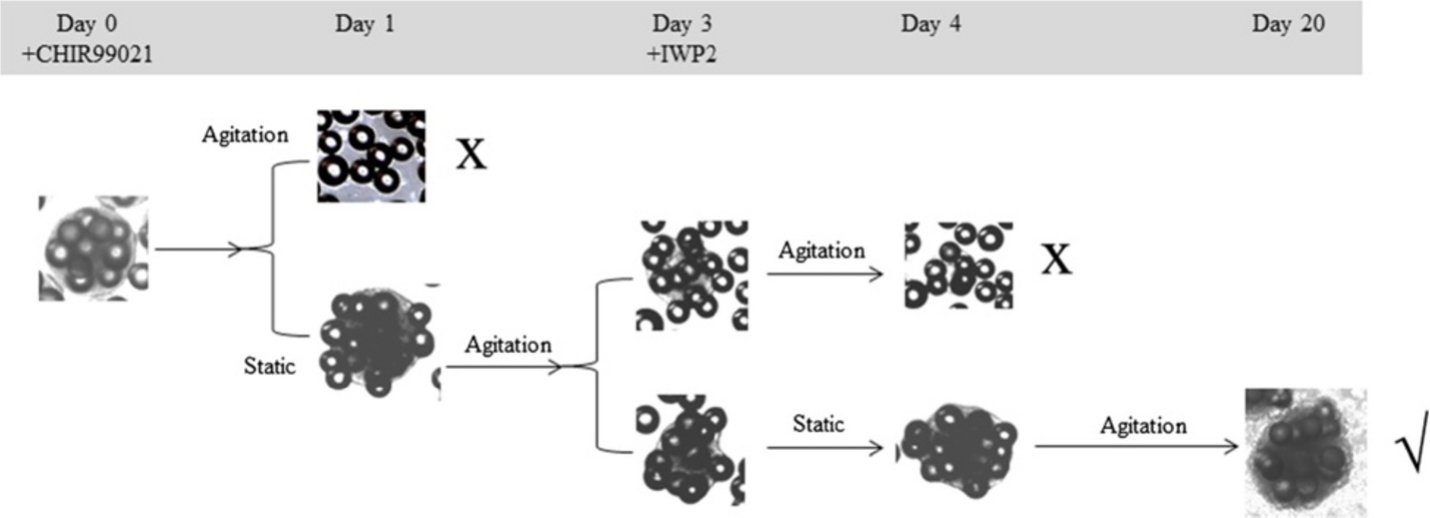
**Effects of different agitation and static modes in the differentiation phase.** Agitation at 110 rpm was applied at different periods of times. X, aggregate disintegration; √, successful aggregate growth.

The same medium was used as in MC-SS with different times for static and agitation (110 rpm) during the period of differentiation, as shown in Figure 2. The successful protocol was static on day 1, agitated on day 2, static again on day 3, and agitated for days 4 to 20.

#### Culture regime 5: hESC/MC aggregates in stirred spinner culture

The best agitation protocol chosen from MC-AS was transposed to culture in a spinner flask. Briefly, hESC/MC aggregates from the exponentially growing hESC MC culture (1.5 × 10^6^ cells/ml) were used. Differentiation was initiated by replacing the spent hESC medium with differentiation medium containing 15 μM CHIR99021. This was incubated in static condition for 16 hours, after which the medium was replaced with fresh differentiation medium (without CHIR99021) and stirred at 30 rpm. On day 3, differentiation medium with 10 μM IWP2 was introduced and incubated with a second static 16-hour pause, followed by agitation from day 4 until the end of the differentiation process (day 20). On day 5 the spent medium was replenished, and refreshed thereafter every 2 days until day 11. The culture was then fed with differentiation medium with insulin every 2 days until day 20. Aliquots (~1 ml) of cell suspension were taken at different time points for gene expression analysis. On day 20, the culture was harvested for structural and functional analyses.

### Fluorescence-activated cell sorting

To test the pluripotency of expanded hESCs, FACS was performed with the expression of the stem cell markers Tra-1-60 (Millipore, Darmstadt, Germany) and mAb84 [34] by flow cytometry as described in our previous studies [33, 35].

CM differentiation efficiency was quantified by the expression of myosin heavy chain (MHC) and cTnT I. Briefly, cells harvested from day 20 were fixed and permeabilised using Fix and Perm Cell permeabilisation reagents (Life Technologies). The cells were subsequently incubated with anti-MHC (dilution 5:200; Developmental Studies Hybridoma Bank, Iowa city, IA, USA) and anti-cTnT (dilution 1:200; Millipore) for 20 minutes. After washing with 1% bovine serum albumin/PBS, the cells were incubated in the dark with 1:500 dilutions of anti-mouse FITC-conjugated secondary antibodies (dilution 1:500; DAKO, Glostrup, Denmark) for 20 minutes in the dark. The signal from labelled cells was acquired using a FACSCalibur and was analysed with FlowJo (Tree Star, Ashland, OR, USA), following the manufacturer’s protocol, with gating selected at the point of intersection between the marker and its isotype control [38].

### Quantitative polymerase chain reaction

Total RNA was isolated from undifferentiated (day 0) and differentiating (at different time points until day 20) hESCs from MNL and hESC/MC aggregates from MC-Sp. This isolation was accomplished using the RNeasy mini kit (Qiagen, Hilden, Germany). The yield and purity of the RNA were determined with a NanoDrop spectrophotometer (NanoDrop Technologies). RNA (1 μg) was reverse transcribed into cDNA via Oligo(dT) with SuperScript III Reverse Transcriptase (Life Technologies). Real-time quantitative polymerase chain reaction was performed on an ABI7500 system (Applied Biosystems, Carlsbad, CA, USA) with Power SYBR Green PCR Master Mix (Applied Biosystems) containing the genes of interest presented in Additional file 2. Thermal cycling was applied as follows: 50°C for 2 minutes, 95°C for 10 minutes, following by 40 cycles of 95°C for 15 seconds and 60°C for 1 minute. Data were analysed and the fold-change of each gene referenced against expression of the same gene prior to cell differentiation.

### Metabolite measurement

Glucose, glutamine, lactate, and ammonia concentrations in the cultures were measured using Bioprofile 100 plus (NOVA, Waltham, MA). Measurements were performed on both spent media and its replacement fresh medium after each medium change. Specific consumption and production rates of the metabolites and yield ratios were calculated as described in Chen and colleagues’ paper [39], by averaging of triplicate experiments.

### Immunocytochemistry

Beating hESCs from MNL and hESC/MC aggregates from MC-Sp were harvested and washed in PBS, and replated in Matrigel-coated 24-well plates (Corning), where they were cultured for 2 days. The cells were then fixed with 4% paraformaldehyde for 15 minutes at room temperature, rinsed in PBS, and permeabilised by incubation with 0.1% Trion X-100/1% bovine serum albumin in PBS for 1 hour at room temperature. They were then blocked for 2 hours in PBS containing 0.1% Triton X-100, 10% goat serum, and 1% bovine serum albumin. Cells were then probed with primary antibodies including anti-sarcomeric α-actinin (anti-SA; Sigma, St. Louis, MO, USA), anti-myosin light chain (Cell Signalling, Danvers, MA, USA), and anti-cTnT (Millipore) for 1 hour, followed by a secondary Alexa Fluor® 594 antibody (Life Technologies) for another 2 hours at room temperature. A fluorescent mounting medium with 4′,6-diamidino-2-phenylindole nuclear staining (Vectashield) was added to cover the cells during their imaging with an Axiovert 200 M fluorescence microscope (Carl Zeiss).

### QT prolongation assay by microelectrode arrays

Beating hESC/MC aggregates were incubated with TrypLE™ Express (Life Technologies) for 30 minutes to dissociate the cells from microcarriers. The cell suspensions were then filtered through a 40-μm cell strainer (Becton Dickinson, San Jose, CA, USA) and placed in an ultra-low attachment U-96 plate (Costar). They were centrifuged at 1500 rpm for 5 minutes and subsequently allowed to form EB-like aggregates for 2 to 3 days until the aggregates resumed beating contractions. The EB-like aggregates were then transferred to ReproCELL Inc., Kanagawa, Japan, who applied their QT prolongation (QTempo) assay on a microelectrode array system. A detailed description of the microelectrode array system is published elsewhere [40, 41]. The results obtained from this system are comparable with the patch clump system [40]. Briefly, the assay consisted of plating the aggregate on a microelectrode array dish (MEA200/30iR-Ti-gr; Multi Channel Systems, Reutlingen, Germany) in QTempo assay medium supplemented with E-4031 or verapamil with increasing concentrations. Each concentration of test compound was added to the beating aggregate every 4 minutes in an accumulative assay method [38, 40]. Two minutes (minutes 0 to 2) were allowed for conditioning, followed by 2 minutes (minutes 2 to 4) for detection, as per ReproCELL’s QTempo assay protocol [40]. Commercially available induced pluripotent stem cell-derived CM (ReproCardio 2) from ReproCELL Inc. was used as positive control. QT intervals (also known as the field potential duration [42]) were determined by measuring the time interval between the beginning of the Q wave and the end of the T wave on the electrocardiogram [43]. The correction of the QT interval for the beating rate is calculated according to Bazett’s formula [44].

**Figure 3:**
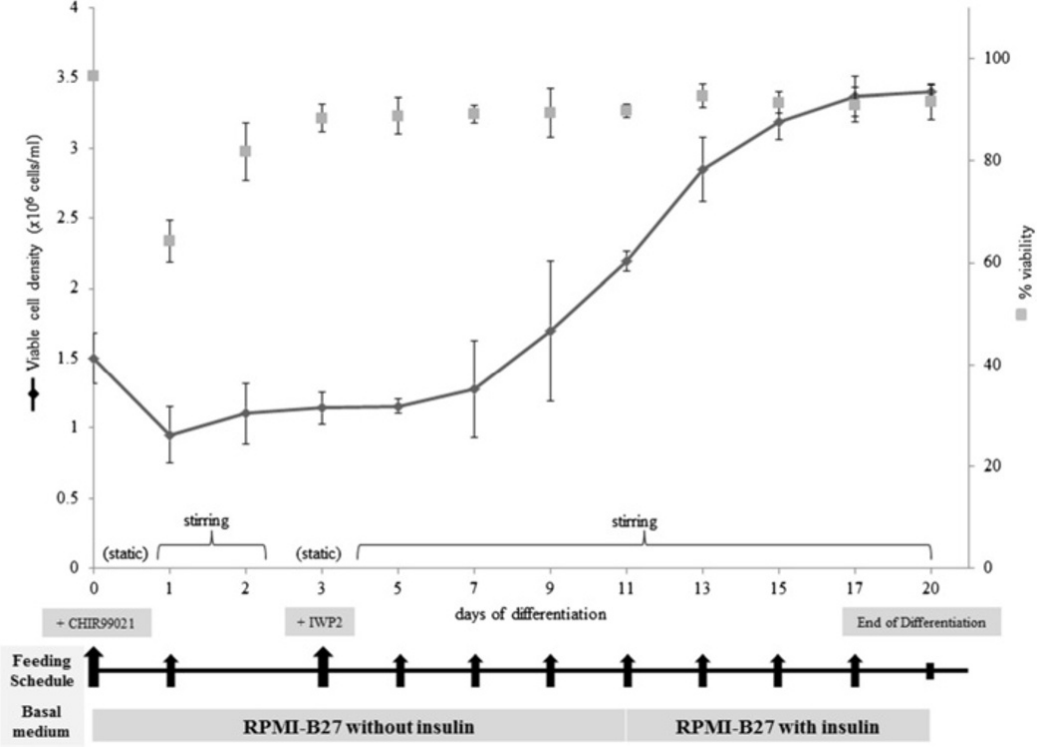
**Growth kinetics of HES3/microcarrier aggregates differentiating in stirred spinner cultures.** The cultures were incubated at 37°C and 5% carbon dioxide in stirring conditions, except on day 1 and day 3 in which the cultures were incubated in static conditions for 16 hours to reduce cell loss due to the addition of Wnt modulators, CHIR99021 and IWP2. Feeding regime as indicated by arrows.

### Statistical analysis

All experiments were performed at least in triplicate. Data values are reported as the mean and standard deviation. Analysis of variance was applied as a comparison between groups, with *P* < 0.05 and *P* < 0.01 considered two levels of statistically significant differences.

## Results

### Differentiation of hESCs to cardiomyocytes via conventional monolayer cultures

HES3 cells were differentiated into CMs using a Wnt modulator-based protocol, as described by Lian and colleagues [12]. The culture matrix was LM rather than Matrigel™, however, in order to develop a defined environment that is suitable for generating and maintaining clinical-grade CMs.

For MNL, the optimal concentrations of CHIR99021 and IWP2 for cardiac differentiation were 12 μM and 5 μM, respectively (see Figure S1A in Additional file 1). Spontaneously contracting regions were readily observed at 8 days’ differentiation. The numbers of contracting foci increased with time, and at day 20 these were observed over almost the entire well (see Additional file 3). These foci appeared to be loosely attached to the plate. On day 20 of differentiation, total cell expansion (cell expansion from seeding the hESC culture in the propagation phase to final CM harvesting in the differentiation phase) was 20 ± 0.8-fold. Furthermore, 45.7 ± 4.6% of cells were MHC-positive and 51.1 ± 0.5% were cTnT-positive as measured by FACS (Table 1). The overall CM yield was 3.8 ± 0.2 CM/hESC, with a final cell concentration of 0.5 ± 0.02 × 10^6^ CM/ml.

**Table 1:** **Propagation and differentiation of HES3 to cardiomyocytes using different culture methods**

Culture conditions	MNL hESC cultures	hESC/MC aggregate spinner cultures			
Propagation (7 days)					
Expansion fold (propagation)	10.3 ± 0.4	15.4 ± 0.4			
% Pluripotent cells					
Tra-1-60-positive	93.1 ± 1.4	96.2 ± 1.7			
mAb84-positive	99.4 ± 0.5	98.6 ± 1.7			
Aggregate sizes (μm)	NA	316 ± 11			
Differentiation to cardiomyocytes (20 days)					
	MNL	MC-Rp	MC-SS	MC-AS	MC-Sp
Expansion fold (differentiation)	2.0 ± 0.2	2.2 ± 0.1	2.1 ± 0.2	2.0 ± 0.2	2.3 ± 0.1
% beating aggregates	Beating sheet	65.7 ± 1.8 (beating foci on plate)	66.5 ± 2.6	53.4 ± 1.5	73.6 ± 2.8
Beating aggregate size (μm)	NA	NA	1063 ± 53	581 ± 54	655 ± 13
% Cardiomyocytes					
MHC-positive	45.7 ± 4.6	43.1 ± 1.2	42.6 ± 1.9	42.8 ± 1.6	47.7 ± 1.9
cTnT-positive	51.1 ± 0.5	53.1 ± 0.9	45.7 ± 2.9	42.4 ± 0.2	56.1 ± 1.4
CM/hESC	3.8 ± 0.2	1.2 ± 0.2	1.0 ± 0.2	0.8 ± 0.1	9.6 ± 0.3^a^
CM/ml (×x10^6^)	0.5 ± 0.02	0.2 ± 0.02	0.1 ± 0.02	0.1 ± 0.01	1.9 ± 0.05^b^
Total expansion fold (propagation + differentiation)	20.0 ± 0.8	32.8 ± 1.1	31.8 ± 2.1	29.3 ± 2.5	34.3 ± 0.9^c^

HES3 cells were propagated as either monolayer cultures (MNL) or human embryonic stem cell (hESC)/microcarrier (MC) aggregates for 7 days. Five differentiation culture regimes were tested. Data presented are mean ± standard deviation (*n* = 3 to 4). In HES3 cultures: ^a^significant difference between MC-Sp and MNL (*P* < 0.05), and between MC-Sp and the other three regimes (*P* < 0.01), in terms of cardiomyocytes produced per initial hESC seeded (CM/hESC); ^b^significant differences (*P* < 0.01) between MC-Sp and the other four regimes in terms of cardiomyocyte density (CM/ml); ^c^significant difference between MC-Sp and MNL (*P* < 0.05) in terms of total expansion fold achieved. CM, number of cardiomyocytes (cTnT-positive cells) in the final cell population; Total expansion fold, cell expansion from seeding the hESC culture in the propagation phase, to final CM harvesting (day 20) in the differentiation phase. cTnT, Cardiac troponin T; MC-AS, hESC/MC aggregates in agitated suspension cultures; MC-Rp, monolayer replated cultures; MC-Sp, hESC/MC aggregates in spinner cultures; MC-SS, hESC/MC aggregates in static suspension cultures; MHC, myosin heavy chain; NA, not available.

### Differentiation of spinner culture expanded hESC/MC aggregates into cardiomyocytes by four different approaches

HES3 cells were first propagated on PLL + LN-coated MCs in mTeSR™1 for 7 days on MC cultures in 50 ml stirred spinner flasks. Similarly to our previous work [35], exponential cell growth started on day 4, with a maximum cell density of ~3 × 10^6^ cells/ml attained at day 7. Cell viability was above 90% (see Figure S2A in Additional file 4), with high expression (>90%) of pluripotent markers Tra-1-60 and mAb84 (Table 1). Spherical hESC/MC aggregates (EB-like), 316 ± 11 μm in size, were generated (Table 1; see Figure S2A in Additional file 4).

After cell propagation, four methods of differentiation for cardiogenesis efficiency were investigated by modulating Wnt [12, 13] – replated hESC/MC aggregates on LN-coated plates (MC-Rp); hESC/MC aggregates in static suspension (MC-SS); hESC/MC aggregates in agitated suspension (MC-AS); and hESC/MC aggregates in spinner cultures (MC-Sp) – as illustrated in Figure 1.

#### Replated hESC/MC aggregates

hESC/MC aggregates attached and subsequently spread within 3 to 4 days, forming a flattened configuration on the LN-coated surface. About 50% of the MCs were released to culture supernatant after cell plating and during differentiation. Beating clusters were first observed as early as day 8, following incubation with 15 μM CHIR99021 and 10 μM IWP2 (see Figure S1B in Additional file 1). On day 20, 65.7 ± 1.8% of beating clusters, lying on beating sheets were scored. Total CM yield was 1.2 ± 0.2 CM/hESC and density was 0.2 ± 0.02 × 10^6^ CM/ml, lower than those from MNL cultures (*P* < 0.05). However, the total expansion was 32.8 ± 1.1-fold, higher than that in MNL (20.0 ± 0.8-fold; *P* < 0.05; Table 1).

#### hESC/MC aggregates in static suspension

Despite the optimal concentrations of CHIR99021 and IWP2 (see Figure S1C in Additional file 1) being used, which were the same as for MC-Rp, beating aggregates were observed later, around day 12 of differentiation. On day 20, the CM density was 0.1 ± 0.02 × 10^6^ CM/ml. Moreover, uncontrolled agglomeration of cell aggregates was observed, resulting in the formation of large aggregates, 1,063 ± 53 μm in size (Table 1). In terms of total expansion, the percentage of beating aggregates scored, and the fraction of MHC-positive and cTnT-positive cells stained, MC-SS yielded similar results to MC-Rp (Table 1).

#### hESC/MC aggregates in agitated suspension

To prevent the formation of very large aggregates during the differentiation phase of MC-SS, agitation at 110 rpm was applied. However, the induced shear stress resulted in massive cell detachment 1 day after adding CHIR99021 (Figure 2). To mitigate this, a static pause (optimally ~16 hours) was introduced after the addition of CHIR99021. This was followed by 2 days of agitation and another static pause (~16 hours) when IWP2 was added, since aggregate dissociation was observed if the culture was continuously agitated (Figure 2). After these two periods, the culture was placed under continuous agitation. At day 20, 53.4 ± 1.5% of aggregates were beating, 42.8 ± 1.6% of cells were MHC-positive, and 42.4 ± 0.2% of cells were cTnT-positive, yielding 0.8 ± 0.1 CM/hESC, and a cell concentration of 0.1 ± 0.01 × 10^6^ CM/ml, similar to the CM yield from MC-SS, but with smaller beating aggregates (581 ± 54 μm) (Table 1).

To summarise, we demonstrated that hESC/MC aggregates can serve as EBs for cardiac differentiation with higher differentiation efficiency. The efficiency between the three culture regimes did not vary to a great extent (0.8 to 1.2 CM/hESC; Table 1). To implement this protocol in bioreactors used for industrial manufacturing, a regime consisting of agitation with intermittent static pauses was introduced. This culture regime retained intact aggregates for differentiation and was applied therefore for direct differentiation in a stirred spinner flask.

#### hESC/MC aggregates in spinner cultures

After demonstrating the feasibility of generating CMs using hESC/MC aggregates in agitated small plates, the next step was to accomplish a conjoint propagation and differentiation process in a stirred spinner flask (MC-Sp). At day 20 of differentiation, this method yielded 73.6 ± 2.8% of beating aggregates (see Additional file 5). Their average size was 655 ± 13 μm, approximately twice the linear dimensions (316 ± 11 μm) of aggregates during the expansion phase (Table 1). FACS analysis showed that up to 47.7 ± 1.9% of cells were MHC-positive and 56.1 ± 1.4% of cells were cTnT-positive. This yielded 9.6 ± 0.3 CM/hESC, about 2.5 times higher than the MNL cultures (3.8 ± 0.2 CM/hESC; *P* < 0.05) and eight to 12 times higher than the MC-Rp, MC-SS and MC-AS (1.2 ± 0.2, 1.0 ± 0.2, and 0.8 ± 0.1 CM/hESC, respectively; *P* < 0.01). A CM concentration of 1.9 ± 0.05 × 10^6^ CM/ml was achieved, about four times higher than the MNL (0.5 ± 0.02 × 10^6^ CM/ml; *P* < 0.01), nine times higher than the MC-Rp (0.2 ± 0.2 × 10^6^ CM/ml; *P* < 0.01), and 19 times higher than the MC-SS and MC-AS (around 0.1 × 10^6^ CM/ml; *P* < 0.01) (Table 1).

#### Cell growth kinetics

**Figure 4:**
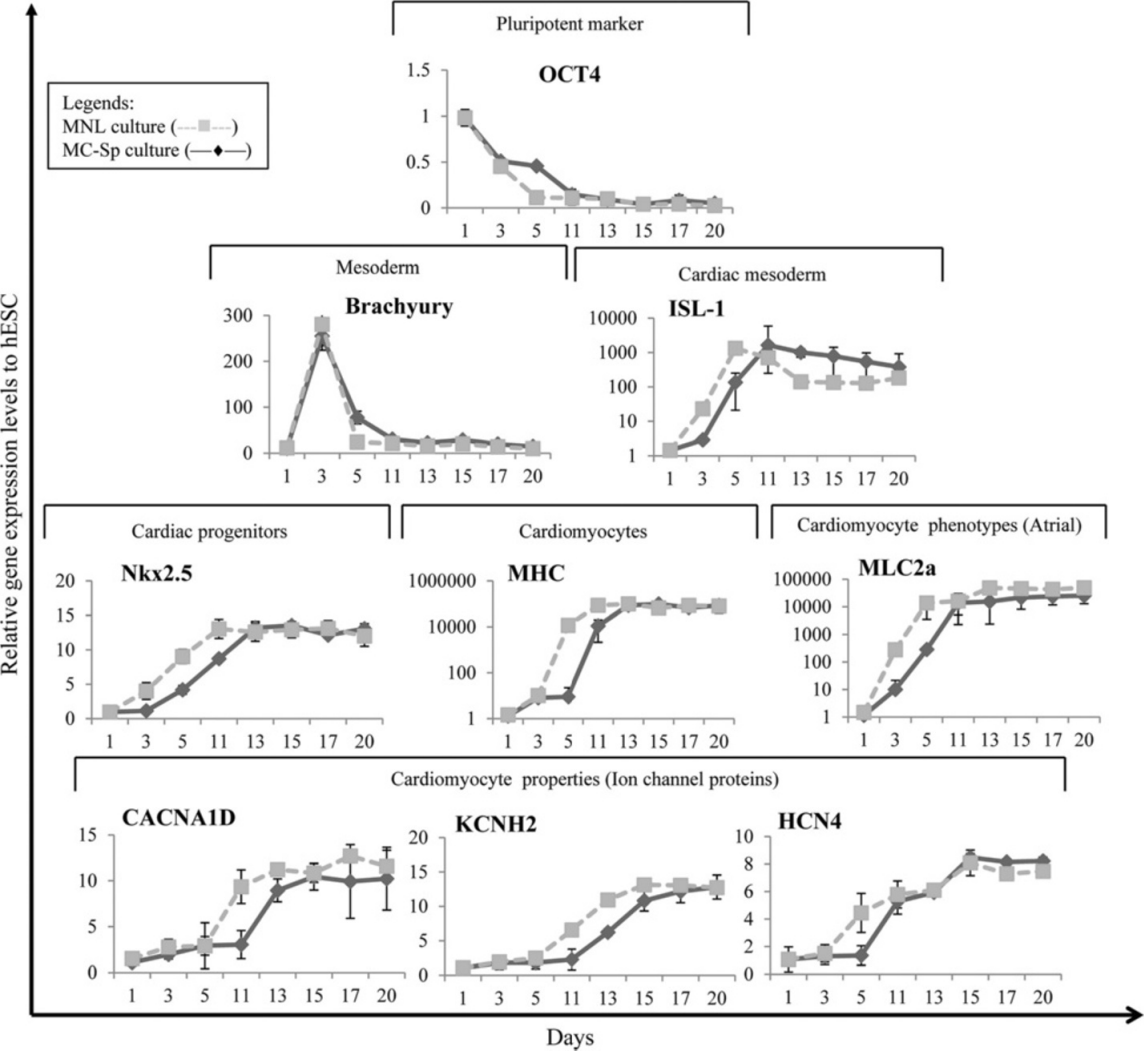
**Temporal gene expression pattern during cardiomyocyte differentiation in monolayer cultures and MC-Sp.** Real-time quantitative polymerase chain reaction data for various hallmark markers of cardiomyocyte differentiation (at days 1, 3, 5, 11, 13, 15, 17, and 20): squares, monolayer cultures (MNL); diamonds, hESC/MC aggregates in spinner cultures (MC-Sp). Data are presented as the relative gene expression levels of differentiated hESCs compared with the undifferentiated state. hESC, human embryonic stem cell; MC, microcarrier.

The kinetics of HES3 cell growth during the differentiation phase is shown in Figure 3. At day 1 of static culture (after addition of CHIR99021), about 30% cell death occurs and cell density gradually increased thereafter. The lag phase, before the onset of rapid cell expansion, lasts about 3 to 4 days. This is similar to that observed for the expansion phase of HES3 on MC in spinner flasks (see Figure S2A in Additional file 4). Concomitantly during this lag phase, lower expression of octamer-binding transcription factor (OCT4) and a higher expression of Brachyury T were observed (Figure 4). This may signal the beginning of differentiation, with lower hESC pluripotency and increasing mesoderm progenitors [45]. The onset of exponential cell growth occurred between days 5 and 7, and maximum cell density (~3.4 × 10^6^ cells/ml) was reached on day 17, followed by a stationary phase in cell expansion. The doubling time was 128 ± 14 hours during the differentiation phase, as compared with 27 ± 2 hours during the expansion phase (Table 2; *P* < 0.001).

**Table 2:** **Doubling times, specific metabolic rates and yield ratios of hESCs in MC-Sp in propagation and differentiation phases**

	HES3 cultures		H7 cultures	
	Propagation	Differentiation	Propagation	Differentiation
Double time (hours)	27 ± 2	128 ± 14	28 ± 2	151 ± 1
Specific consumption and production rate (mmol/10^9^ cells/hour)				
qGlc	0.36 ± 0.04	0.24 ± 0.02	0.34 ± 0.05	0.18 ± 0.02
qLac	0.63 ± 0.12	0.32 ± 0.02	0.57 ± 0.08	0.23 ± 0.01
qGln	0.036 ± 0.003	0.033 ± 0.001	0.028 ± 0.006	0.029 ± 0.003
qAmm	0.029 ± 0.003	0.023 ± 0.002	0.019 ± 0.005	0.016 ± 0.002
Yield ratio				
Y_Lac/Glc_	1.72 ± 0.14*	1.34 ± 0.04*	1.66 ± 0.01*	1.27 ± 0.06*
Y_Amm/Gln_	0.80 ± 0.02	0.70 ± 0.04	0.67 ± 0.03	0.55 ± 0.01

Glucose (Glc), lactate (Lac), glutamine (Gln), and ammonia (Amm) were measured using Bioprofile 100 plus. Specific consumption and production rates of the metabolites (q_m_) were expressed as number of moles of metabolite consumed or produced per cells in one hour. Yield ratio (Y_a_/_b_) was calculated by the value of q_a_ over q_b_. *P < 0.05 indicated there is a significant difference between the values of Y_Lac_/_Glc_ in the propagation phase and the differentiation phase in both HES3 and H7 cultures. MC-Sp, human embryonic stem cell/microcarrier aggregates in spinner cultures.

A second hESC cell line, H7, was used to test this conjoint propagation (see Figure S2B in Additional file 4) and differentiation platform (see Additional file 6). H7 cells propagated on PLL + LN MC cultures achieved cell yields of ~2.3 × 10^6^ cells/ml (see Figure S2B in Additional file 4) or 12-fold cell expansion, generating hESC/MC aggregates with dimensions of 418 ± 17 μm (see Additional file 7). H7/MC aggregates subsequently differentiated into CMs showed a similar differentiation pattern to HES3, yet achieved a cell density of ~3 × 10^6^ cells/ml (see Additional file 6), with a doubling time of 151 ± 1 hours (Table 2). H7/MC aggregates during the differentiation phase were larger than the HES3/MC aggregates (Table 1 vs. Additional file 7). The size of the aggregates may influence the differentiation efficiency [27, 29, 38], giving rise to a final output of 6.6 ± 0.4 CM/hESC initially seeded in the culture and a cell concentration of 1.3 ± 0.09 × 10^6^ CM/ml (see Additional file 7), lower than those obtained for HES3 MC-Sp (Table 1).

#### Metabolic measurements

Data for the consumption of glucose and glutamine as well as the production of lactate and ammonia were measured for HES3 and H7 in the expansion and differentiation phases of MC-Sp, as shown in Table 2. The metabolic activity of both hESC lines was considerably lower during the differentiating phase, with respect to their expansion phase. The molar ratio of produced lactate to consumed glucose, Y_Lac_/_Glc_, is almost 2 (1.72 ± 0.14) during the HES3/MC expansion phase. This indicates that cell respiration was mostly anaerobic [46], as compared with the differentiation phase, where a lower Y_Lac_/_Glc_ (1.34 ± 0.04) suggests that cells tend to generate ATP through oxidative phosphorylation via aerobic metabolism. In contrast, values of Y_Amm/Gln_ in both the expansion and differentiation phases were similar (0.80 ± 0.02 and 0.70 ± 0.04, respectively), which fall within a range expected for mammalian cells (0.7 to 0.84) [39, 46]. Similar results were observed for H7 MC-Sp.

#### Molecular and structural characterisation

**Figure 5:**
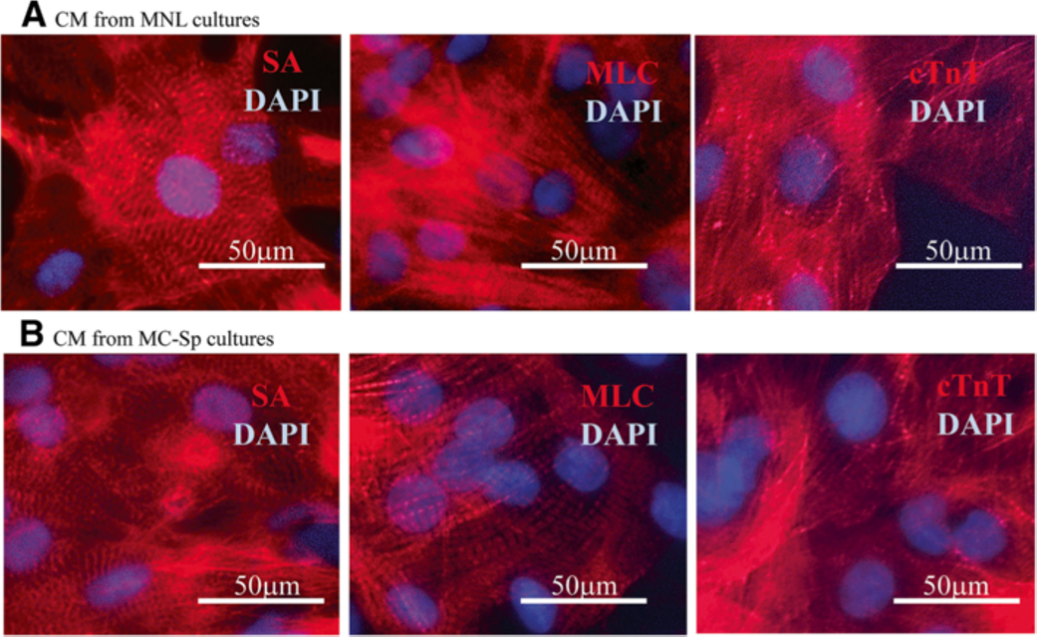
**Immunostaining of structural proteins in monolayer cultures and MC-Sp.** Immunostaining of structural proteins in **(A)** monolayer cultures (MNL) and **(B)** hESC/MC aggregates in spinner cultures (MC-Sp). Nuclei were stained with 4′,6-diamidino-2-phenylindole (DAPI) (blue). Scale bar represents 50 μm. CM, cardiomyocytes; cTnT, cardiac troponin T; hESC, human embryonic stem cell; MC, microcarrier; MLC, myosin light chain; SA, sarcomeric α-actinin protein.

The dynamics of gene expression in hESC/MC aggregates, during the differentiation phase of MC-Sp, and in hESCs from differentiation MNL were monitored by quantitative polymerase chain reaction. The cardiac gene expression profile of the CMs derived from MNL (Figure 4) is similar to the profile reported in the literature [12]. The kinetic trends of all gene expression are similar in both MNL and MC-Sp. However, a delay in the decline of the pluripotency marker and an increase of cardiac mesoderm as well as progenitor gene expression was observed in MC-Sp. The onset of CM differentiation was marked by a decrease in the pluripotency marker, OCT4, following the replacement of culture medium with differentiation medium (containing CHIR99021) at day 0. This marker decreased to negligible levels by day 11. The expression of mesoderm marker, Brachyury T, was transient. It peaked on day 3 and returned to its baseline expression by day 11. This simultaneous decrease in OCT4 and increase in Brachyury T was observed during the lag phase (day 3 to day 5) of cell growth (Figure 3). A subsequent increase in expression of the cardiac mesoderm marker Islet-1 and early cardiac marker homeobox protein Nkx-2.5 began on day 3. This expression then reached its peak at day 11 and saturated on day 13. Thereafter, the relative expression of late cardiac markers such as α-MHC was also significantly upregulated from day 5 and persisted throughout the 20 days’ differentiation. A high expression of myosin light chain-2 atrial, which reached its maximum on day 11, indicated the presence of atrial CMs. The expression of cardiac ion channel proteins (CACNA1D, encoding the α1D subunit of the L-type calcium channel; KCNH2, the potassium voltage-gated channel; and HCN4, the potassium/sodium hyperpolarisation-activated cyclic nucleotide-gated channel 4, responsible for the pacemaker current) was also significantly upregulated after day 11 of the differentiation phase.Immunofluorescence analyses were performed to determine the presence of cardiac-specific proteins at day 20. The hESC monolayer-derived CMs (Figure 5A) and hESC/MC-derived CMs (Figure 5B) both stained positive for sarcomeric proteins – α-actinin, myosin light chain, and cTnT – thus showing a well-organised sarcomeric structure, a phenomenon associated with maturing CMs.

#### Functional characterisation

**Figure 6:**
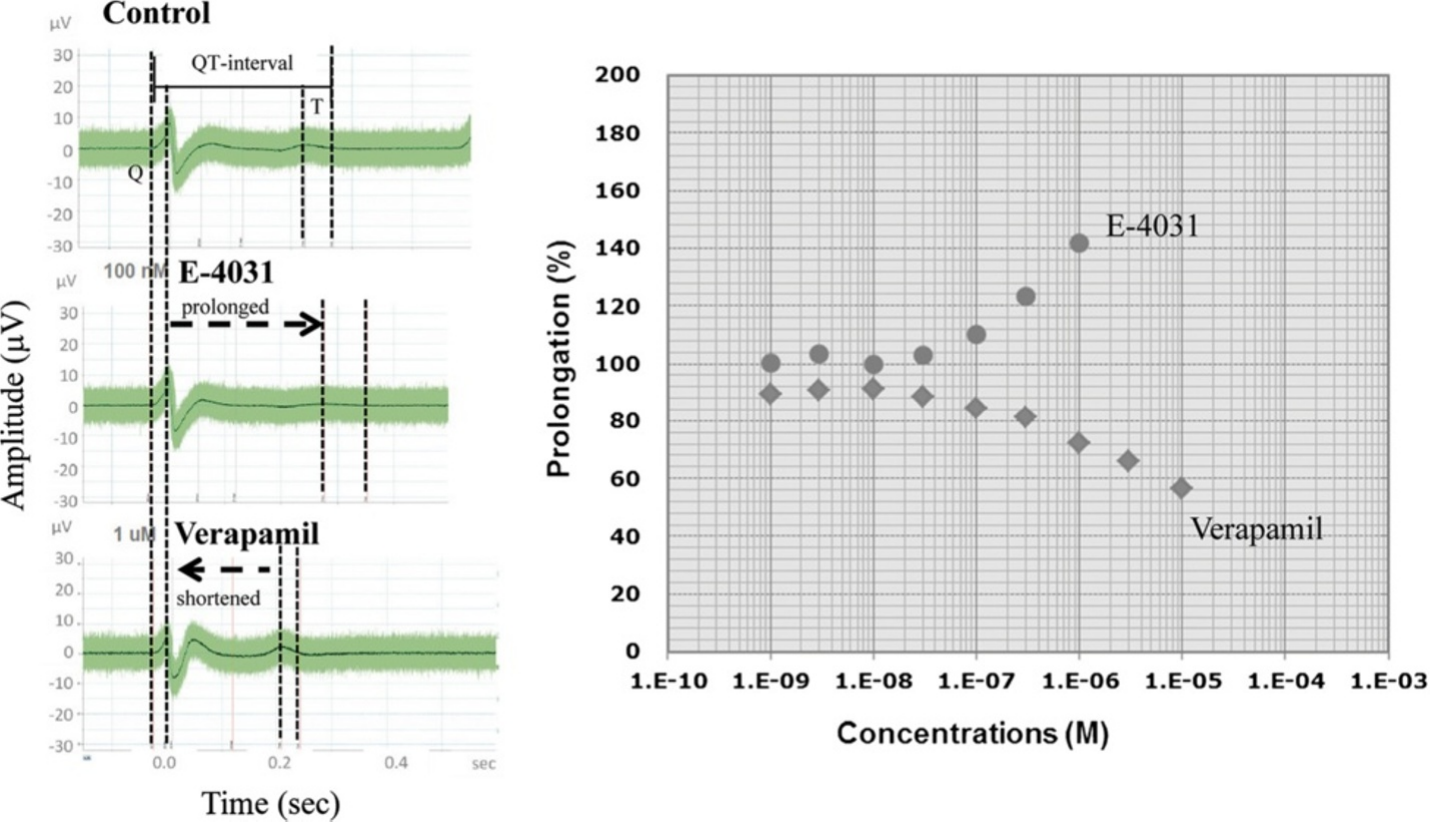
**Dose-dependent effect of E-4031 and verapamil on duration of the QT interval of differentiated MC-Sp cardiomyocytes.** Dose-dependent effect of E-4031 (•) and verapamil (♦) on duration of the QT interval of differentiated MC-Sp cardiomyocytes using the QTempo assay (conducted by ReproCELL Inc., Kanagawa, Japan). Increase in E-4031 concentration from 10 to 300 nM results in prolongation of the QT interval by 40%, while increase in verapamil concentration from 10 nM to 3 μM results in reduction of the QT interval by 56%. MC-Sp, human embryonic stem cell/microcarrier aggregates in spinner cultures.

Beating CMs from cultures at day 20 were seeded onto a multielectrode plate, which served to test their response to pharmacologically active compounds. When the selective hERG inhibitor E-4031 was introduced, a dose-dependent prolongation of the QT interval was observed from a threshold of 10 nM, peaking at 300 nM (rising to over 140% from 100%; Figure 6). In contrast, verapamil (L-type calcium channel blocker) induced a dose-dependent reduction of the QT interval at concentrations ranging from 10 nM to 3 μM to 56% of the starting condition (Figure 6). Similar responses were observed using the induced pluripotent stem-derived cell line ReproCardio 2 (see Additional file 8). It is important to note that the QT-interval duration is directly proportional to the action potential duration in cardiomyocytes [42, 43, 47]. These results demonstrated that hESC/MC-derived CMs generated by MC-Sp responded normally [40] to these anti-arrhythmic drugs.

## Discussion

A recent review by our group discussed in detail the key issues for large-scale production of human CMs from hPSCs [31]. One of the limitations of differentiating hPSCs to CMs is the low cell expansion, twofold to fivefold [31, 48]. This is similar to our data from the present study, showing about twofold expansion (Table 1; see Additional file 7). The strategy for CM production would thus be biphasic, involving hESC propagation to generate significant cell numbers, followed by differentiation to CMs. Two hESC propagation platforms (Figure 1) were used in this study: monolayer and MC/aggregate hESC cultures. Conventional MNL have been widely employed for hESC expansion, but the increase in cell density is restricted to the available culture area [31, 34]. MCs are characterised by high surface-to-volume ratio, which allows for high-density cell culture, and the possibility of scaling up has been demonstrated by several groups using spinner flasks [31, 34, 49, 50]. The present study demonstrates that MC spinner cultures achieved 15.4-fold pluripotent HES3 expansion (similar to our previous study [35]), which is fivefold more than those achieved in MNL (Table 1). There is further room for improvement in this scalable process through controlled feeding strategies to obtain higher cell expansion [36].

Having established a scalable hESC propagation method, a suitable CM differentiation platform should be developed [31]. The present report compares five modes of differentiation, where the CM/hESC yield was ranked as follows: MC-Sp >> MNL > (MC-Rp ≈ MC-SS) > MC-AS (Table 1). The conjoint propagation and differentiation protocol (MC-Sp) eliminates the labour-intensive cutting, size control, and replating that are required by EB culture methods [26–28]. hESC/MC aggregates (about 300 to 400 μm in size) obtained during the propagation phase are subjected to a differentiation medium by continuing in the same culture vessel. A simple medium change thus induces the CM differentiation process from HES3 to generate cTnT-positive CMs (9.6 ± 0.3 CM/hESC in MC-Sp vs. 3.8 ± 0.2 CM/hESC in MNL; *P* < 0.05) and the concentration of CMs obtained (1.9 ± 0.05 × 10^6^ CM/ml) was fourfold higher than from MNL (0.5 ± 0.02 × 10^6^ CM/ml; *P* < 0.01) (Table 1).

Moreover, the total cell expansion (from seeding hESC culture to final CM harvesting) in this integrated system was about 34.3-fold, whereas in MNL the expansion was 20-fold (*P* < 0.05; Table 1). These differences are mainly attributed to the three-dimensional nature of the aggregate cultures as compared with the one-layer two-dimensional nature of the MNL. The MC-based method lends itself easily to scalable bioreactor processing, in contrast to planar MNL, which require complex stacking arrangements and are thus poorly suited for scalable processes. hESC/MC-derived CMs from MC-Sp cultures were observed to express cardiac markers associated with cardiomyogenesis (Figures 4 and 5B), demonstrating a similar trend to that observed in hESCs differentiating on MNL (Figures 4 and 5A) and followed the cardiac developmental process [51, 52]. This MC-based conjoint propagation and differentiation system is thus overall more efficient than MNL for the high-yield production of CMs in a reliable and robust process.

Prior studies demonstrated the significant role of uniform-sized cell aggregates in differentiation outcomes [27, 29, 38]. Reports from Zandstra’s group suggested that the optimal aggregate size ranges from 200 to 800 μm, where the highest percentage of contracting aggregates is produced by 400 μm aggregates [53, 54]. Another study showed that the generation of 300 μm aggregates, using microwells, was the most efficient method for producing contracting cells [29]. Studies by our group also suggested that a size distribution of 200 to 400 μm generally gives higher cell expansion and more MHC^+^/SA^+^ cells [38]. However, that same study generated hPSC/MC aggregates in spinner cultures from MNL, following a differentiation protocol using SB203580, a mitogen-activated protein kinase inhibitor. In short, another advantage of the present MC-based system is its ability to generate aggregates with specific sizes (316 μm in HES3, Table 1; and 418 μm in H7, see Additional file 7). More importantly, the ability to control the size of an aggregate within a scalable bioprocess may contribute to a more homogeneous [35], synchronised differentiation [27, 29, 30].

A decline in cell density and viability was observed following the initial exposure of both HES3/MC and H7/MC aggregates to CHIR99021, in MC-Sp (Figure 3 and see Additional file 6). The observed cell loss may be attributed to the abrupt change in culture medium, from mTeSR™1, to differentiation medium (RPMI/B27), as well as the toxicity of CHIR99021, when used at high concentrations (>10 μM) [55]. This cell loss could be alleviated when static pauses were employed (Figure 2), in order to prevent additional agitation-induced cell death due to further stress arising from turbulent eddies, MC-to-MC impacts and MC-to-container (or impeller) impacts [56]. Dissociation of cells/MC aggregates were also observed after addition of IWP2 (Figure 2). Studies showed that IWP2 blocks Wnt protein secretion, leading to the proteasome-mediated degradation of β-catenin; and this is detrimental to the adherent junctions in cell–cell adhesion complexes [57, 58]. A second static pause (~16 hours) was thus introduced to mitigate the possibility of cells dislodgement from the MC due to agitation forces. In a nutshell, these two static pauses successfully maintained a stable increase in the viable cell density during the differentiation phase (Figures 2 and 3; see Additional file 6).

Although agitated MC-AS and MC-Sp are both amenable to control the size of the differentiating cell aggregates, lower cell yields were obtained in MC-AS (0.1 vs. 1.9 × 10^6^ CM/ml, *P* < 0.01; Table 1). This is attributed to the high agitation speeds of the plates on a shaking platform (110 rpm to suspend hESC/MC aggregates) potentially generating higher hydrodynamic forces than the gentle agitation by an impeller, operating at lower speed (30 rpm), in stirred flasks [59]. This putatively resulted in poorer hESC growth and differentiation efficiency, as measured in MC-AS [60]. Moreover, cells can be seeded at 10-fold higher density (~1.5 × 10^6^ cells/ml) in MC-Sp, than the inoculum cell density in MNL, MC-Rp, MC-SS, or MC-AS (~1.5 × 10^5^ cells/ml). In addition, the well-mixed environment of MC-Sp is better for delivering nutrients and oxygen to MC cell aggregates via convection of the fluid medium [59, 61]. High cell viability is thus maintained without compromising the differentiation efficiency, resulting in higher CM yields. The present study further confirms previous work from our group, describing that microcarrier cultures facilitate higher pluripotent and differentiated cell (neuroprogenitors) yields [32, 34–36].

Quantification of the nutrient uptake and respiration products offers evidence that the hESC energy metabolism differs between the propagation and differentiation phases. In the hESC propagation phase, the higher Y_Lac/Glc_ indicates anaerobic glycolysis (Table 2). Reports state that stem cells favour anaerobic glycolysis for cell survival and proliferation, but not differentiation, since they only possess immature mitochondria for energy supply in order to control the stem cell state [62, 63]. In contrast, lower Y_Lac/Glc_ (1.27 to 1.34; Table 2) during cell differentiation suggests that this phase is accompanied by an increase in the aerobic metabolism of the cells. Reports have shown that differentiation events are associated with the proliferation of mitochondria, which generate more energy for the molecular machinery in response to differentiation [64, 65]. Another study has demonstrated that the inhibition of Wnt signalling facilitates mitochondrial respiration, with induction of the glycolytic switch into an aerobic metabolism [66]. This correlates with the findings of the present study.

This platform may potentially be improved to obtain higher yields and purity of CM cells for clinical applications; for example, by altering the feeding regime to daily or twice-daily medium exchange [36]. Moreover, the addition of ascorbic acid has been demonstrated to promote cardiac differentiation and has been applied in several protocols, using hESCs and human induced pluripotent stem cells [14, 67]. To implement good manufacturing practice production criteria, human LM could replace murine LM for propagating and differentiating hESCs in a serum-free, xeno-free, defined environment. Depending on the size of the bioreactor, larger quantities of clinical-grade CMs can thus be generated to meet therapeutic dose requirements.

## Conclusion

Five methods of differentiation were compared and MC-Sp were the best condition for scale-up and production of hESC-derived CMs, within an integrated process of cell propagation and subsequent differentiation. This MC-based conjoint culture is crucial to achieving both high cell densities (1.9 × 10^6^ CM/ml) and high CM/hESC yields (9.6 ± 0.3), as compared with the other tested methods. CMs expressed cardiac-specific transcriptional factors, structural and functional genes, and generation of cross-striated muscle structure that recapitulate the development ontogeny of cardiogenesis. The advantages of this approach are: high cell yields; controlled aggregate size; negligible labour-intensive manual intervention (time-saving); and use of cost-effective defined chemical components (a potential cost-saving), which has the potential to comply with a defined good manufacturing practice bioprocess system. The scalable nature of this MC-based bioprocess under agitation is expected to provide a platform technology for the bioreactor-based production of CMs derived from hPSCs.

## Electronic supplementary material

Additional file 1: **Is a figure showing optimization of the concentrations of Wnt modulators, CHIR99021 and IWP2, in MNL (A); MC-Rp (B); and MC-SS (C).** Cells were analysed for MHC and cTnT expression by flow cytometry 20 days after initiation of differentiation. The optimal concentrations are marked in the white box. Step 1: optimization of CHIR99021 concentration (IWP2 concentration kept at 5 μM); Step 2: optimization of IWP2 concentrations (the optimal concentrations of CHIR99021 were used in Step 1). (TIFF 1 MB)

Additional file 2: **Is a table presenting the sequences of primers used for quantitative polymerase chain reaction.** (TIFF 113 KB)

Additional file 3: **Is a video showing HES3 cultured as a monolayer on a LN-coated plate (MNL) and treated with 12 μM CHIR99021 at day 0 and 5 μM IWP2 at day 3 in RPMI/B27 medium without insulin.** Video 1 shows cardiomyocytes from day 20. (WMV 2 MB)

Additional file 4: **Is a figure showing growth kinetics of MC stirred spinner cultures of (A) HES3 and (B) H7 during the propagation phase.** H7 produced larger aggregates than HES3 in PLL + LN MC spinner flask. (TIFF 126 KB)

Additional file 5: **Is a video showing integrated propagation and differentiation of HES3 in MC culture platform (MC-Sp).** Video 2 shows cardiomyocytes from day 20. Round spheres inside the cell clumps are the MCs (diameter ≈ 100 μm). (WMV 1 MB)

Additional file 6: **Is a figure showing growth kinetics of H7/MC aggregates differentiating in stirred spinner cultures.** The cultures were incubated at 37°C and 5% carbon dioxide in stirring conditions, except on day 1 and day 3, in which the cultures were incubated in static conditions for 16 hours to reduce cell lost due to the addition of Wnt modulators, CHIR99021 and IWP2. Feeding regime is as indicated by arrows. (TIFF 99 KB)

Additional file 7: **Is a table presenting integrated propagation and differentiation of H7 to cardiomyocytes in MC spinner cultures.** (TIFF 95 KB)

Additional file 8: **Is a figure showing the dose-dependent effect of E-4031 (A) and verapamil (B) on duration of the QT interval of ReproCardio 2 induced pluripotent stem cell-derived cardiomyocytes using the QTempo assay (conducted by ReproCELL Inc.).** Results are presented as actual measurements (•) and after correction with Bazett (■) or Fredericia (▲) formulas. Increase in the E-4031 concentration results in prolongation of QT intervals, while increase in the verapamil concentration results in reduction of QT intervals. (TIFF 3 MB)

## Abbreviations

CM: cardiomyocytes (cardiac troponin T-positive cells) in the final cell population
cTnT: cardiac troponin T
EB: embryoid body
FACS: fluorescence-activated cell sorting
hESC: human embryonic stem cell
hPSC: human pluripotent stem cell
IWP: inhibitor of Wnt production
LN: laminin
MC: microcarrier
MC-AS: hESC/MC aggregates in agitated suspension cultures
MC-Rp: monolayer replated cultures
MC-Sp: hESC/MC aggregates in spinner cultures
MC-SS: hESC/MC aggregates in static suspension cultures
MHC: myosin heavy chain
MNL: monolayer cultures
OCT4: octamer-binding transcription factor 4
PBS: phosphate-buffered saline
PLL: poly-l-lysine
SA: sarcomeric α-actinin.
