# Supplementary figures and images for: Conjoint propagation and differentiation of human embryonic stem cells to cardiomyocytes in a defined microcarrier spinner culture
Source: Stem Cell Res Ther. 2014 Sep 15;5(5):110. doi: 10.1186/scrt498 (PMC4183116; doi:10.1186/scrt498)

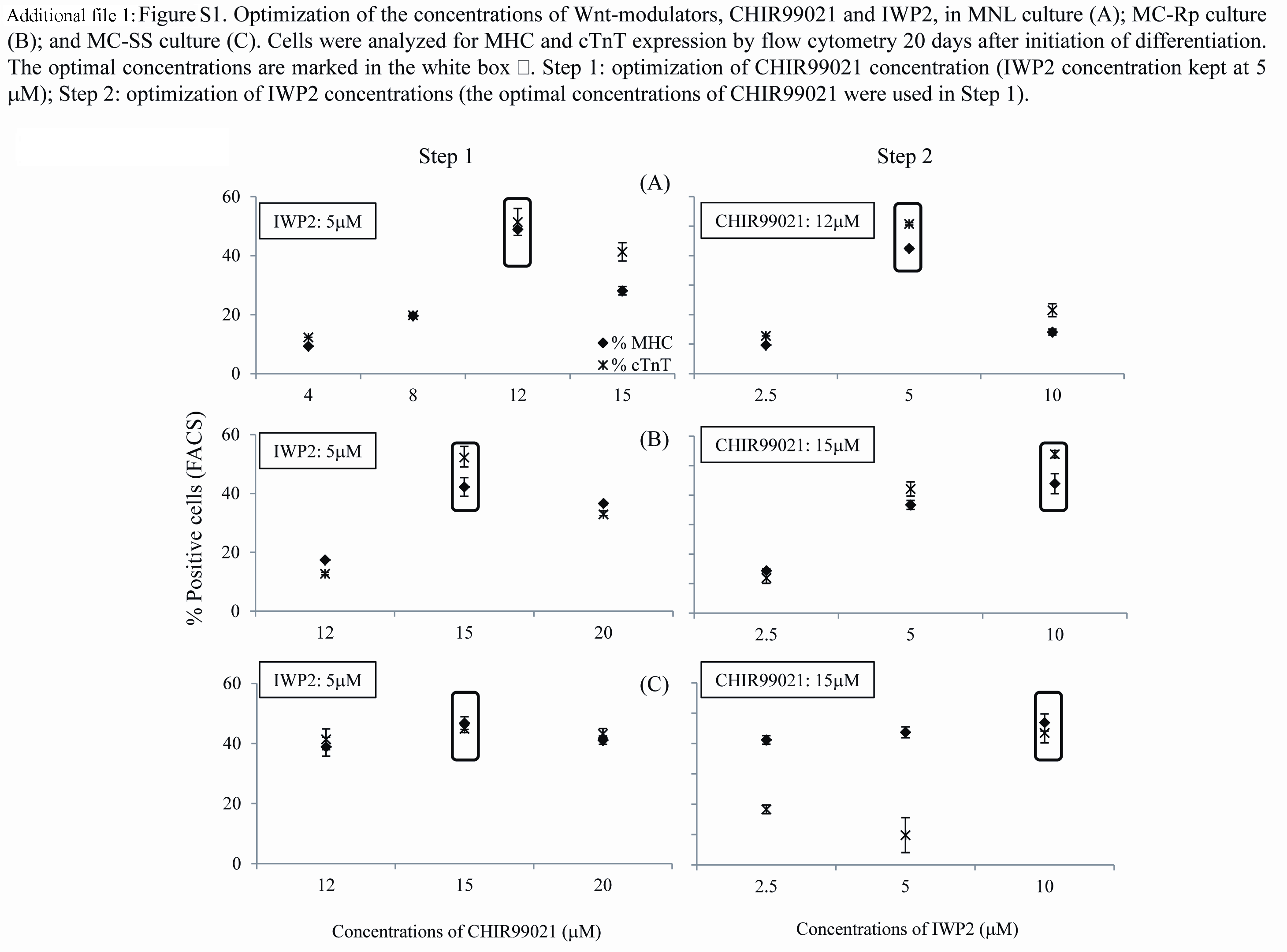

Supplement: Supplementary file 1 — Additional file 1: Is a figure showing optimization of the concentrations of Wnt modulators, CHIR99021 and IWP2, in MNL (A); MC-Rp (B); and MC-SS (C). Cells were analysed for MHC and cTnT expression by flow cytometry 20 days after initiation of differentiation. The optimal concentrations are marked in the white box. Step 1: optimization of CHIR99021 concentration (IWP2 concentration kept at 5 μM); Step 2: optimization of IWP2 concentrations (the optimal concentrations of CHIR99021 were used in Step 1). (TIFF 1 MB) [file 13287_2014_395_MOESM1_ESM.tiff]

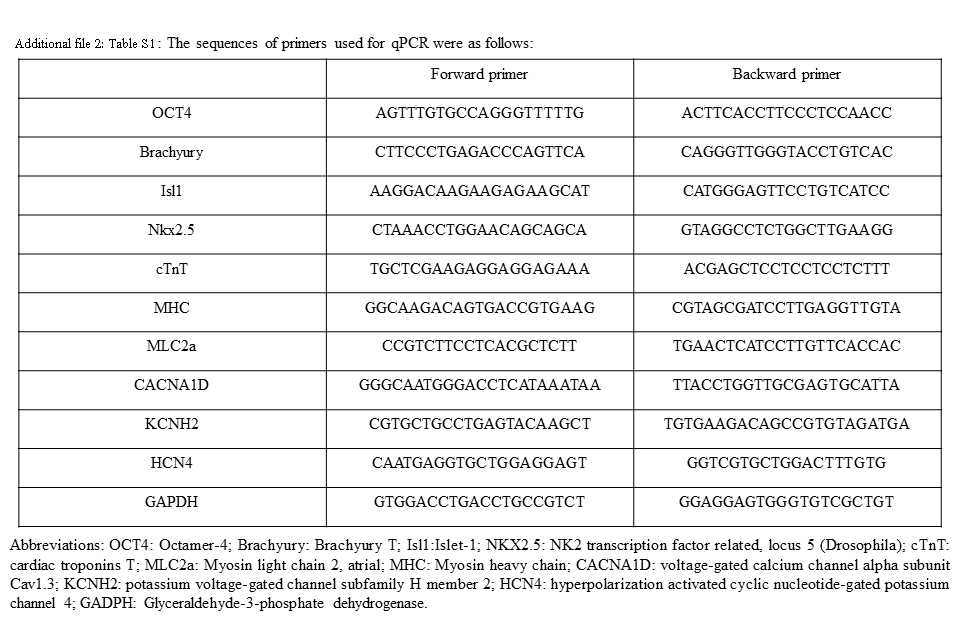

Supplement: Supplementary file 2 — Additional file 2: Is a table presenting the sequences of primers used for quantitative polymerase chain reaction. (TIFF 113 KB) [file 13287_2014_395_MOESM2_ESM.tiff]

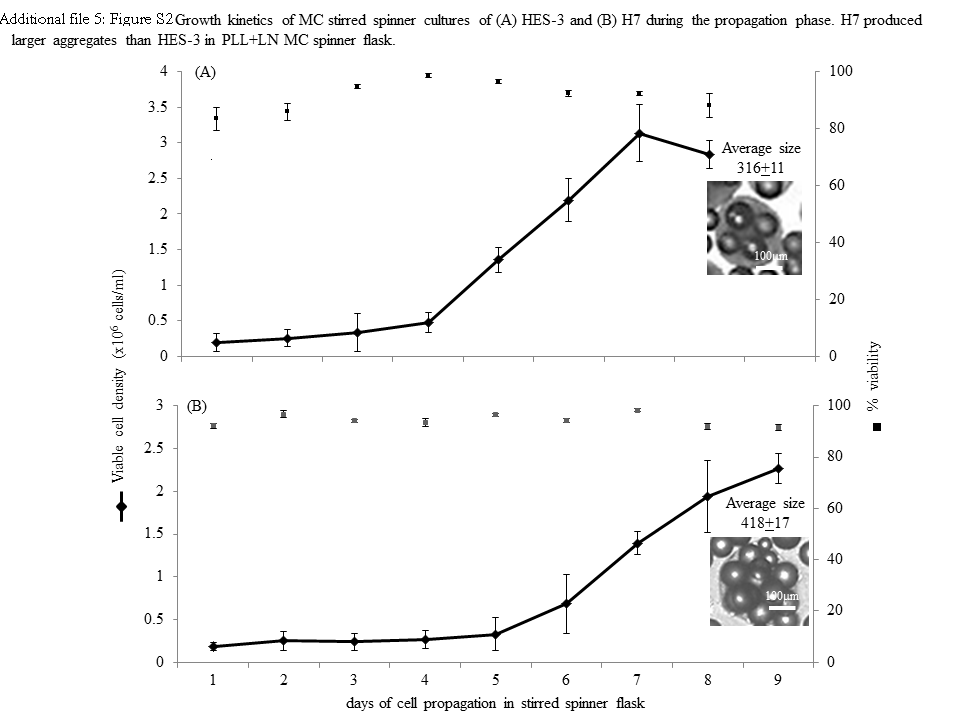

Supplement: Supplementary file 4 — Additional file 4: Is a figure showing growth kinetics of MC stirred spinner cultures of (A) HES3 and (B) H7 during the propagation phase. H7 produced larger aggregates than HES3 in PLL + LN MC spinner flask. (TIFF 126 KB) [file 13287_2014_395_MOESM4_ESM.tiff]

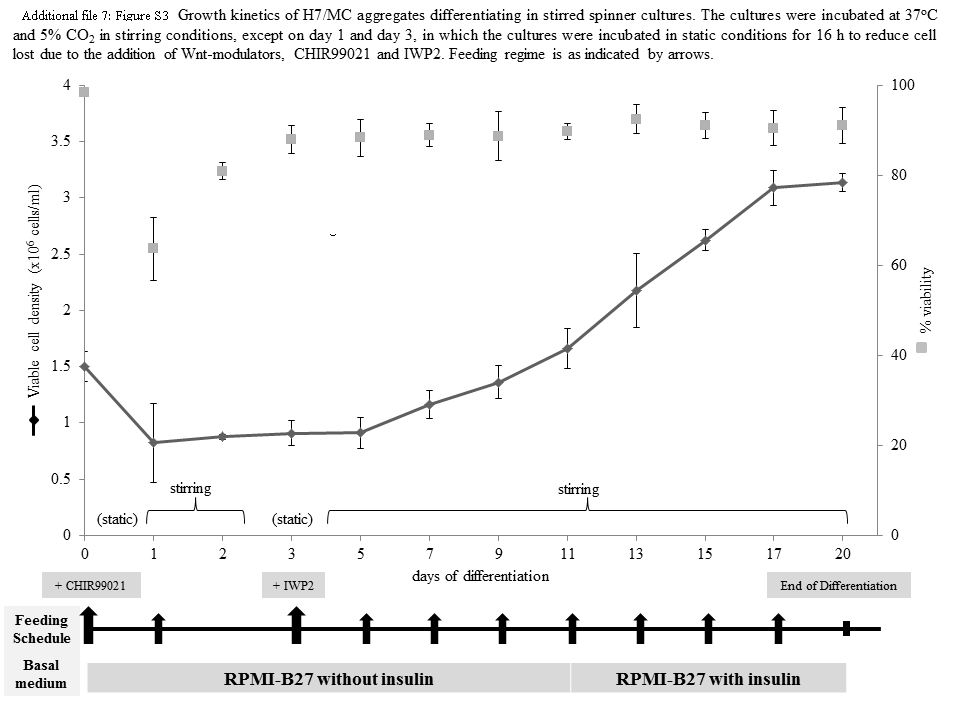

Supplement: Supplementary file 6 — Additional file 6: Is a figure showing growth kinetics of H7/MC aggregates differentiating in stirred spinner cultures. The cultures were incubated at 37°C and 5% carbon dioxide in stirring conditions, except on day 1 and day 3, in which the cultures were incubated in static conditions for 16 hours to reduce cell lost due to the addition of Wnt modulators, CHIR99021 and IWP2. Feeding regime is as indicated by arrows. (TIFF 99 KB) [file 13287_2014_395_MOESM6_ESM.tiff]

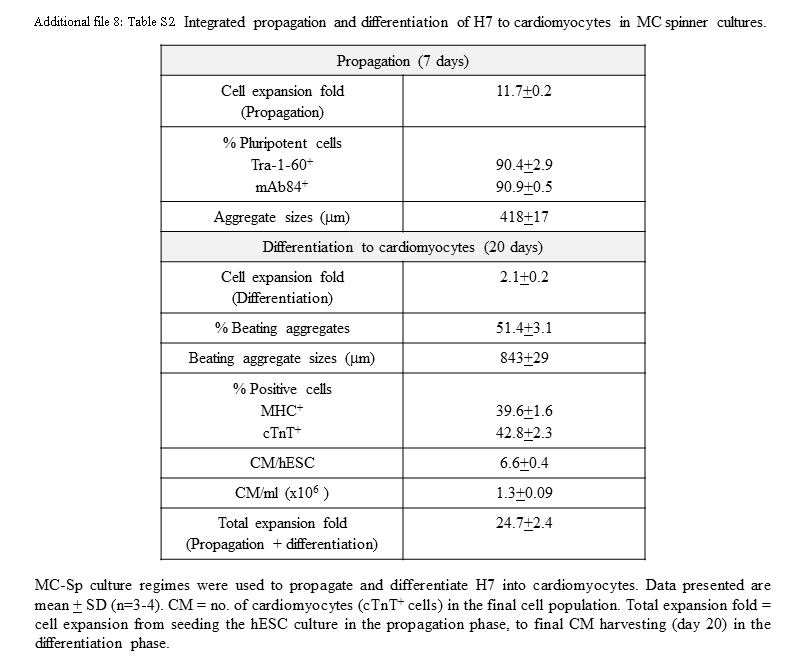

Supplement: Supplementary file 7 — Additional file 7: Is a table presenting integrated propagation and differentiation of H7 to cardiomyocytes in MC spinner cultures. (TIFF 95 KB) [file 13287_2014_395_MOESM7_ESM.tiff]

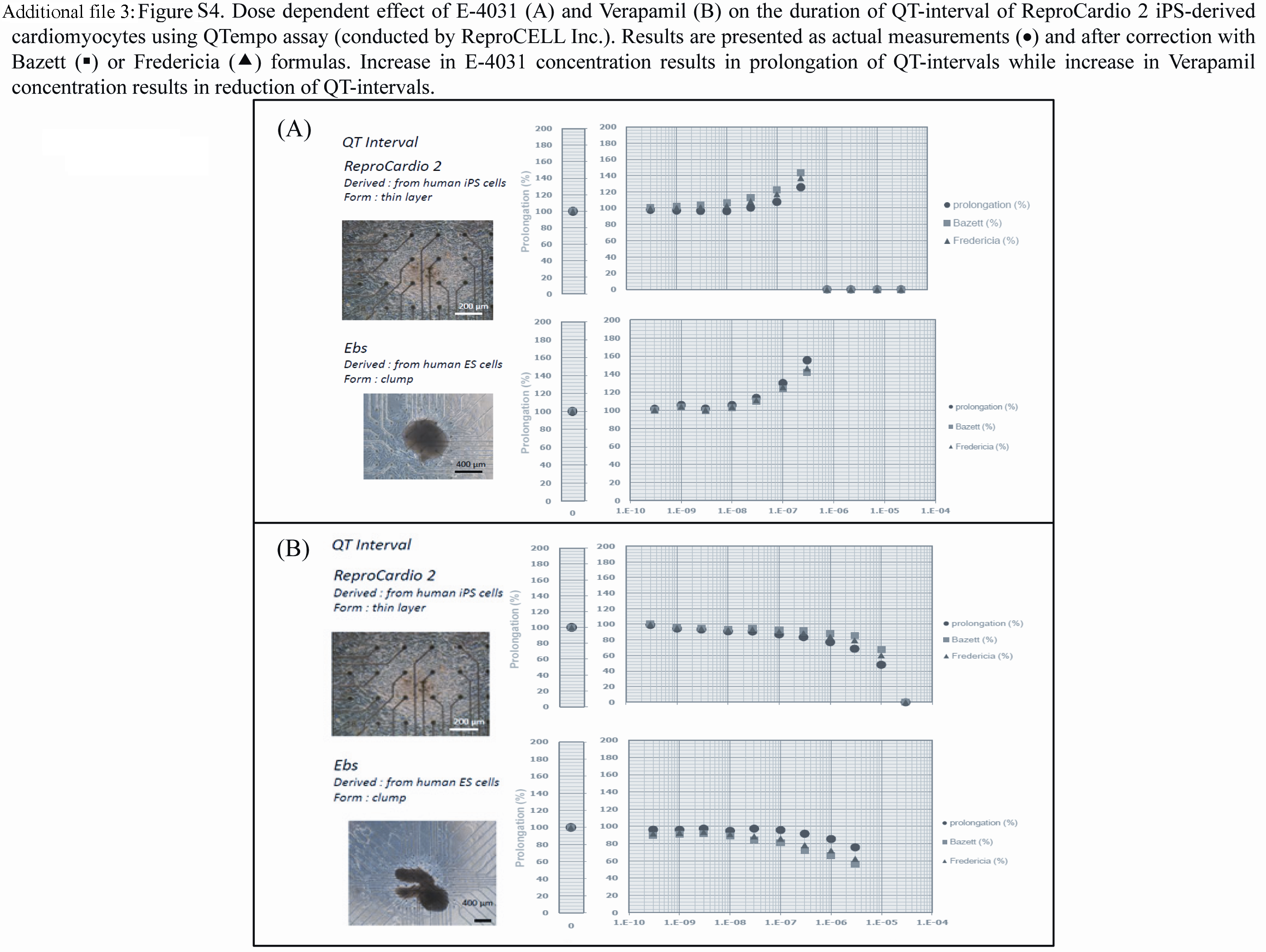

Supplement: Supplementary file 8 — Additional file 8: Is a figure showing the dose-dependent effect of E-4031 (A) and verapamil (B) on duration of the QT interval of ReproCardio 2 induced pluripotent stem cell-derived cardiomyocytes using the QTempo assay (conducted by ReproCELL Inc.). Results are presented as actual measurements (•) and after correction with Bazett (■) or Fredericia (▲) formulas. Increase in the E-4031 concentration results in prolongation of QT intervals, while increase in the verapamil concentration results in reduction of QT intervals. (TIFF 3 MB) [file 13287_2014_395_MOESM8_ESM.tiff]
